# The Patterns of Intraspecific Variations in Mass of Nectar Sugar along a Phylogeny Distinguish Native from Non-Native Plants in Urban Greenspaces in Southern England

**DOI:** 10.3390/plants12183270

**Published:** 2023-09-14

**Authors:** Kowiyou Yessoufou

**Affiliations:** Department of Geography, Environmental Management and Energy Studies, University of Johannesburg, P.O. Box 526, Auckland Park, Johannesburg 2006, South Africa; kowiyouy@uj.ac.za

**Keywords:** functional traits, invasion biology, nectar production, native plants, non-native plants, urban greenspaces

## Abstract

To serve human needs, non-native species are selected based on an array of functional traits, which generally confer competitive advantages to these species in their recipient environments. Identifying non-obvious functional traits that indirectly inform human selection of non-natives to introduce into urban greenspaces is not yet part of common discussions in invasion biology. We tested whether functional traits integrated within a phylogenetic framework, may reveal those subtle criteria underlying the introduction of non-native plants into urban greenspaces. We found no differences in terms of functional traits between natives and non-natives. We also found no evidence that functional traits predict nectar production, irrespective of how nectar production was measured. Finally, we found that the mean sugar concentration of nectar per flower is evolutionarily shared both within closely related non-native plants as well as within close native plants. However, phylogenetically close species share similar intraspecific variation in mass of nectar sugar per flower, but this is true only for non-native plants, thus revealing a non-obvious selection criteria of non-native plants for urban greenspaces. Our results indicate that the phylogenetic patterns of intraspecific variation in mass of nectar sugar per flower is the major criterion distinguishing non-natives from native plants in urban greenspaces in Southern England.

## 1. Introduction

Species are moved across the globe accidentally or intentionally by humans [1] for specific ecosystem services these species provide, e.g., horticultural, medicinal, or ecological services [2,3]. However, although introducing non-native species into new environments provides some benefits (e.g., erosion control, medicinal uses, etc.), their introductions also raise environmental concerns [1]. One of those concerns is the naturalization and then invasion of these species [4]. Then two important questions to ask are (i) what are the functional traits that predispose non-native species to naturalization? and (ii) in the scenario of intentional selection and introduction, what are the contributions of human, as the selection agent of those species, to this naturalization? The first question is widely explored [5]. Indeed, almost 60 years ago, Baker [6] deployed great efforts to document functional traits that predispose non-native plants to weediness and invasion (see also [5,7]). However, the second question still deserves attention. A recent study demonstrated that non-native plants of economic values (e.g., animal food and ornamental) are almost 20 times more likely to naturalize than not, and the naturalization likelihood increases for non-native plants that provide higher number of uses ([3]; see also [2]), thus showing the importance of human selection criteria of non-native plants in contributing to their naturalization. I define human selection criteria as the reasons (e.g., erosion control, medicinal uses, food, horticultural values, etc.) underlying the decision by humans to intentionally move a species beyond its native range. In the present study, I referred to these intentional reasons as ‘obvious selection criteria’ which are determined by some plant functional traits. I argued that there are other selection criteria, apart from the obvious ones, that I termed ‘hidden or non-obvious criteria’. I defined hidden criteria as those that are not part of the criteria that inform the intentional selection by humans of a given non-native plant for introduction into a new range, but which play critical roles in the success of non-native plants in their introduced ranges. As such, hidden criteria may include phylogeny, nectar production, etc. Ref. [3] showed a phylogenetic pattern in the naturalized flora, thus implying that human selection criteria of non-native plants have a phylogenetic component. These studies [2,3] provide evidence that the understanding of obvious (e.g., horticultural and medicinal) or hidden criteria (e.g., phylogeny) subtending human selection of non-native species is key for predicting their outcomes (e.g., naturalization) or the mechanisms that provide an upper hand to non-native species in the competition for ecological dominance. 

Various strategies through which non-native species are conferred competitive advantages over natives are widely reported [8,9]. For example, the ability to adjust their flowering phenology and thus their interactions with pollinators confer such advantages to non-native species. Non-native species tend to flower over longer periods compared to natives [5] or shift their flowering period to track a changing climate [10]. Non-native species are also reported to co-opt pollinators of native species [11], a strategy which will eventually lead, in the long run, to the decline of the population of native plant species [12]. This co-option is facilitated by the development of a suite of traits, e.g., large and durable floral displays, copious nectar, and pollen rewards [11], which represent key innovations promoting the fitness and invasion of non-native species. The fitness of alien plants into a new environment requires the development of key strategies to co-exist with native species [13], and a pre-requisite for such co-existence for some non-native plants is a successful competition over native species for pollinators [11,14].

Indeed, the introduction of non-native species into a new environment is disruptive to the established ecological networks, including alterations in the flow of nutrients and energy [15] and biotic interactions [9,16,17], resulting in the establishment of new selection pressures [18,19,20]. Flowers are one of plant’s organs actively used in the realization of biotic interactions, energy flow, and selection pressures (see [14]). One of the functional attributes of flowers is the production of nectar, suggesting that any adjustment to the flowering phenology would affect the nectar production. Consequently, we expect that non-native plants, flowering for an unusually longer period [5] or shifting their flowering phenology [10] to co-opt the pollinators of native plants [11], may eventually produce more nectar than native plants. The rationale for this expectation is that, given the ongoing unprecedented decline in insect pollinators worldwide and particularly in England [21], producing higher volume of nectar (than natives) may be a strategy to recruit and satisfy the needs of more pollinators (that are now becoming rare [10]), which then might eventually prefer high-nectar-producing non-native plants to natives. In support of this hypothesis, evidence exists showing that pollinators, e.g., hummingbirds which are obligate nectar feeders, have developed an ability to distinguish between high and low nectar-producing plants across the landscape [22,23]. This means that, in the scenario where non-native plants produce more nectar than native plants, hummingbird-pollinated non-native plants would be favored for pollination. 

Unfortunately, most of the available data on nectar production are from natural systems [24,25,26], making it impossible to investigate whether nectar production is a part of non-obvious or hidden criteria that inform human selection of non-native species that are to be introduced into a new environment. Interestingly, data on nectar production in man-made systems such as urban greenspaces are now emerging [27,28]. Indeed, in the context of an ever-declining population of pollinators [21], urban greenspaces are increasingly appreciated as hotspots of pollinators diversity [29,30]. 

Urban greenspaces are public or private open spaces comprising all sorts of greenery, including parks, green roofs, woodlands, community gardens, lawns, sporting fields, ornamental plant arrangements, etc., which form an urban ecological system for a sustainable city [31,32,33]. Urban greenspaces are acknowledged as extremely beneficial to human health conditions [34,35] and therefore represent a study model of interest. Their salutogenic benefits to humans are diverse, ranging from low mortality and morbidity in the context of pandemics [35,36,37], increased mental wellbeing [37,38,39], and clean air [40]. The mechanisms underlying these health benefits may be linked to human direct usages of greenspaces [34]. For example, the exposure to greenspaces boosts the protective activities of natural killer cells [41], and they protect humans against infections [42]. Also, species composition of the urban greenspaces may also be critical in their effects on human health. For example, positive effects of urban greenspaces to humans have been linked to the presence of essential oil-producing plants [43,44]. Since urban greenspaces are man-made ecosystems—humans determine their species composition—understanding the selection criteria of species, e.g., functional traits, as well as the origins (native vs. non-native plants) of species to compose the urban greenspaces becomes a pre-requisite for a well-informed design of urban greenspaces that would be fully beneficial not only to the environments but also to human health conditions. 

Another important function of urban greenspaces that is not well debated is their nectar production services, which is linked to the diversity of pollinators in urban areas. In a recent study, ref. [27] showed that, although urban greenspaces do not produce quantitatively more nectar than farmland and nature reserves, the origin of nectar supply in greenspaces is more diverse, driven mostly by non-native plants. Specifically, compared with other systems, urban gardens account for 85% of urban plant nectar supply [27], but it remains unclear whether nectar supply can be detected as ‘hidden or non-obvious’ criteria underlying human selection of non-native plants.

In the present study, the aim was to determine non-obvious criteria informing the selection of non-native plants by humans. To this end, we compared native and non-native plants from different angles, expecting to determine those non-obvious criteria. Specifically, we first investigated if there are significant differences between natives and non-natives in terms of functional traits. Then, we asked the following question: do plant functional traits (life-forms, origins, and floral traits) predict nectar production? Finally, we tested if phylogeny predicts nectar production in both native and non-native plants. These three questions provide opportunities for comparative analysis between native and non-natives, allowing to identify the suite of criteria that inform human selection of non-native plants to be included in urban greenspaces. Identifying such criteria may shed light on human contribution to the selection of the functional traits that predispose non-native species to naturalization and eventually to invasion.

## 2. Results

First, we investigated if there are significant differences between natives and non-natives in terms of functional traits. We found no such differences (life-forms: χ^2^ = 1.88, df = 2, *p* = 0.39; floral structures: χ^2^ = 3.29, df = 11, *p* = 0.98). 

Second, we asked the following question: do plant functional traits (life-forms, origins, and floral traits) predict nectar production? Fitting a phylogenetic ANOVA, we found no such evidence, irrespective of how nectar production was measured. For example, plant origins do not correlate with nectar production measured either as Nectar_mass_SD (Holm–Bonferroni corrected *p* = 0.797), Sugar_per_FU_ug (Holm–Bonferroni corrected *p* = 0.60), or Nectar_mass_mean (Holm–Bonferroni corrected *p* = 0.89). Also, plant life-forms do not predict nectar production, Nectar_mass_SD (*p* = 0.826), Sugar_per_FU_ug (*p* = 0.569), and Nectar_mass_mean (*p* = 0.856), and neither do floral structures, Nectar_mass_SD (*p* = 0.658), Sugar_per_FU_ug (*p* = 0.078), and Nectar_mass_mean (*p* = 0.771).

**Figure 1:**
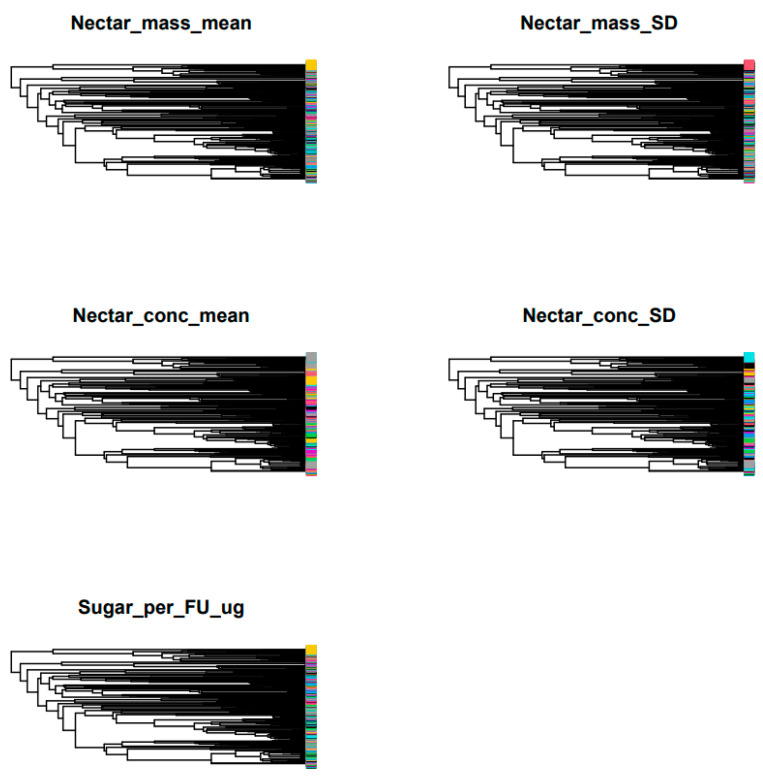
Illustrations of phylogenetic signal in nectar production. A repeat of the same color along a phylogeny for several clades means a repetition of same value of nectar production, implying phylogenetic signal. Nectar_mass_mean = daily mean mass of nectar sugar per flower; Nectar_conc_mean = the mean sugar concentration of nectar per flower; Nectar_conc_SD = standard deviation of the mean sugar concentration (intraspecific variation); Sugar_per_FU_ug = mass of nectar sugar per floral unit; and Nectar_mass_SD = standard deviation of the mean mass (intraspecific variation).

Finally, we tested if phylogeny predicts nectar production. We found evidence supporting this, but only for Nectar_mass_SD and Nectar_conc_mean (Figure 1) whether all species (natives + non-natives) are combined (Nectar_mass_SD: K = 0.22, *p* = 0.003; Nectar_conc_mean: K = 0.18, *p* = 0.03; Table 1) or considered separately: non-native only (Nectar_mass_SD: K = 0.22 *p* = 0.004; Nectar_conc_mean: K = 0.18, *p* = 0.03; Table S1) or native only (Nectar_conc_mean: K = 1.06, *p* = 0.04, Table S2).

## 3. Discussion

The attraction of pollinating insects by plants depends on certain functional traits including floral traits, suggesting that if non-native species lure or co-opt the pollinators of native species [11,13], the flowers of non-native plants may be structurally or functionally different from those of native plants. We would therefore expect floral traits to help distinguish between native and non-native. We found no significant differences between native and non-natives in terms of functional traits such that life-forms, and floral structures do not predict species origin, that is, cannot be used to differentiate between native and non-native plants in urban greenspaces (floral trait similarity). This finding has several implications. The floral trait similarity between non-native and native plant species implies that both species may be sharing similar pollinators (flower visitor overlap; [45]). One consequence of such visitor overlap is an interspecific pollen transfer between native and non-native plants and vice versa [46]. This pollen transfer may lead to reproductive failure due to incompatibility of pollens with ovaries ([47]; but see [48]), and native plants are reported to be the most negatively affected by such reproductive failures, ultimately leading to the decline in the long term of the native plant populations (see [49]). 

Another consequence of floral trait similarity between non-native and natives is strong competition of both species for pollinators (competitive exclusion principle), which may eventually lead to native plant species losing pollinators to non-natives (see [11,12,13,14]). However, such dramatic expectation, i.e., loss of pollinators, was not observed in the study of Ref. [45], but they did report a stronger competition. In the context of the ongoing decline of pollinators populations [21], which is putting the survival of native plants at risk, increasing opportunities for pollinators of native plants to be shared with non-natives may further heighten this risk. Instead, introducing into urban greenspaces non-native plants that do not share similar floral traits with natives, that is, who would not be competing strongly with native plants for pollinators [45], is most preferrable as this would be an opportunity for an increased diversity of pollinators in the urban areas.

Furthermore, the absence of significant differences in floral structures between native and non-native plants is likely driven by humans, that is, humans may select and introduce non-native species that preferentially exhibit a suite of traits that are similar to those of the native species. If that is the case, then this would be evidence of non-random selection of non-native plants (see nonrandom hypothesis; [2]). Such non-random selection of plants by humans for the services they provide is widely proven for native species (e.g., [50]), but not so for non-natives (except in a handful studies; [2,51,52]). However, most of those studies demonstrated a nonrandom introduction of non-native species for medicinal purposes. The present study provides additional evidence that the nonrandom selection of non-native species could actually be based on floral traits (indirectly on nectar production services), which is indicative of an indirect human mediation of plant–insect interactions as well as an indirect human mediation of native–non-native plant interactions. 

Why does the identification of services for which humans select and introduce non-natives plants into a new environment matter? One of the reasons is that services are linked to the invasion ability of non-native plants, and this is based on the following rationale. Functional traits are linked to the services that plants provide and at the same time correlate with the invasion status of species [5]. Therefore, we should expect services to also correlate with the invasion outcome of non-native plants [52]. Such relationships between services and invasion have been tested and supported, but indirectly [5,11]. Furthermore, we should also expect non-native plants providing multiple services to be strong candidates for multiple independent introductions in various quantities into new environments [53,54,55]. Such non-native species are more predisposed to invasion as predicted in the propagule pressure theory [53]. The question then is the following: is nectar production a good candidate for services informing human selection of non-native plants? Although we have evidence of pollinators distinguishing plants that produce more nectar from those producing low quantity of nectar [22,23,24], the contribution of nectar production to species invasion is not yet debated in the invasion biology discourse. The first step taken to initiate such a debate is to determine if selection of non-native plants to introduce is also driven by humans’ direct or indirect preference of nectar production services that these plants provide. In a recent study, Yessoufou and Ambani [52] demonstrated that non-native woody plants providing either medicinal, food or fuel services to humans are more likely to be naturalized in South Africa. How about nectar production?

Evidence of interspecific variations in the quantity of nectar production is well established [27,28,56,57], ranging from 0.1–10 µL (for standing crops) to >650 µL (for columnar cacti and agaves; e.g., [56]). This interspecific variation is interpreted as adaptive strategy of flowering plants to pollinators [24]. For example, adaptive evolution or co-evolution has been invoked between flowers and pollination by hummingbirds, leading to the production of nectar of specific sugar concentrations [58]. Such adaptive measures imply that closely related plant species share specific pollinators, similar competitive ability for pollinators, food preferences, etc. [59]. As such, we would expect a phylogenetic signal in interspecific nectar production in flowering plants. In an early study, Ornelas et al. [60] tested whether phylogeny and flowers’ traits (e.g., flower size) correlate with interspecific variations in nectar production of hummingbird-visited plants. They demonstrated a significant phylogenetic signal in nectar volume and its sugar concentration. They further demonstrated evidence of adaptive measures between nectar and sugar productions with corolla length [60]. In the present study, we found no evidence of functional traits predicting nectar production, irrespective of how nectar production was measured. Specifically, we found that neither plant origins, plant life-forms, nor floral structures predict any of the metrics of nectar production. If any of the plant functional traits does not correlate with nectar production, this means that the criteria used by humans to select horticultural plants for urban greenspaces are similar across all plants, and this similarity drives similar nectar production. If that is the case, we should then expect a significant phylogenetic signal in interspecific nectar production. 

Unlike Ornelas et al.’s [60] study, our analysis failed to detect a significant phylogenetic signal in interspecific variation in nectar production. However, we found evidence of phylogenetic signal in the intraspecific variation in mass of nectar sugar per flower (Nectar_mass_SD) and in the mean sugar concentration of nectar per flower (Nectar_conc_mean) whether all species (natives + non-natives) are combined, or only non-native plants are considered separately. For native plants, the signal was found only in Nectar_conc_mean. Nectar_mass_SD is indicative of the intraspecific variation in mass of nectar sugar per flower, and as such, the phylogenetic signal that we found in Nectar_mass_SD suggests that closely related species share similar intraspecific variation in mass of nectar sugar per flower but this evolutionarily shared intraspecific variation in mass of nectar sugar per flower is found only for non-native plants. This signal may mean two things. First, it means strong adaptations of flowering plants to pollinators in terms of nectar mass sugar production. Second, this is also a strong evidence of nonrandom selection of the non-natives by humans that they introduce into their urban greenspaces. However, the mean sugar concentration of nectar per flower is too evolutionarily shared, both within closely related non-native plants as well as within close native plants. Nectar characteristics (production and concentration) were reported to show high inheritability [61,62], and this is supported by our finding of significant phylogenetic signal (see also [25,26]). These nectar characteristics are known to correlate with floral traits [60,63,64] and are under strong pollinator-mediated selection [65]. 

In invasion biology, the question of what predisposes non-native plants to naturalization and invasion is a widely investigated question. Some of these investigations report that some functional traits (e.g., phenology, height, seed production, etc.) provide an upper hand to non-native species in terms of competitive ability, thus predisposing them to naturalization and invasion [5]. These traits are the same traits that are linked to the services and goods that humans are after, resulting in selection of non-native species that they introduced into a new environment [2,3]. This means that if traits of interests linked to the services non-native plants provide to human can be identified, one can predict what may predispose non-natives to naturalization and invasion [2,3]. However, while some of these traits are obvious because they are intentionally selected by humans, others are hidden, that is, are not part of the initial set of traits humans are after (e.g., phylogenetic relatedness, nectar production, etc.). Those hidden traits, e.g., phylogeny, play an important role in driving naturalization and invasion (see Darwin Naturalization Hypothesis; [5]). Our aim was to determine non-obvious criteria informing at least indirectly the selection of non-native plants by humans. The findings indicate that none of the functional traits considered are unique to non-natives, and none of them predicts nectar production of native and non-native plants. However, phylogenetically close species share similar intraspecific variation in mass of nectar sugar per flower, but this is true only for non-native plants, thus revealing human hidden selection criteria of non-native plant selection for urban greenspaces. Altogether, our results indicate that the phylogenetic patterns of intraspecific variation in mass of nectar sugar per flower is the major criterion distinguishing non-natives from native plants in urban greenspaces in Southern England. We therefore suggest that such intraspecific variation is potentially one of the functional traits predisposing non-native species to naturalization and invasion in England.

Overall, by showing that some aspects of nectar production distinguish native plants from non-native plants introduced into urban gardens, this study first pointed to potential roles that nectar production played in human selection of non-native plants but also suggest that nectar production services may be a potential criterion for multiple introductions in different numbers of non-natives. As predicted in the propagule theory, such non-natives may eventually become naturalized and invasive in their recipient environment. This hypothesis warrants to be tested to eventually clarify the potential role of nectar production in invasion biology. What we already know is that at country [52] or at global scale [3], non-natives providing multiple or unique economic services are more likely to naturalized in their introduced environments. The present study suggests that differences in patterns of nectar production not only influence human selection criteria of non-native species that can be selected and introduced into urban greenspaces but also could be, pending further investigations, good candidates for ecological services predisposing non-natives to naturalization and invasion in recipient environments.

## 4. Materials and Methods

### 4.1. Study Area

Ref. [28] collected and published the data analyzed in the present study in Southern England in 2018–2019 in Ashley Down allotment, Brackenwood Plant and Garden Centre, Didcot town, Royal Horticultural Society Garden Wisley, Speldhurst village, University of Bristol Botanic Garden, University of Bristol Halls of Residence, and University of Bristol Royal Fort Gardens, UK. Specifically, the data were collected in public and private gardens, allotments, garden centers, hedges, and road verges as well as flower meadows in urban greenspaces such as ornamental borders and shrubberies, lawns, paths, and hard standings (see further details in [28]).

### 4.2. Data Collection

Data analyzed in the present study were retrieved from ref. [28] and comprised 225 flowering plants sampled between 2018 and 2019 in Southern England’s urban greenspaces. 

First, we retrieved the taxonomic details of the plants, including species and families but also their origins (native or non-native to British Isles), as well as their functional traits, e.g., life-forms (shrub, herb or climber), and floral traits (single flower, single capitulum, secondary umbel, single raceme, single thyrse, single compound cyme, single cyme, single corymb, single branch of capitula, and part of panicle). Second, we retrieved data on nectar production by 225 flowering plants from ref. [28] that sampled plants at two to three locations. Prior to nectar measurement, ref. [28] prevented insects’ interactions with the flowers by mesh bags for 24 h and then measured nectar either directly from flowers (102 taxa) or indirectly (123 taxa) by rinsing nectaries with distilled water. Nectar measurements include the daily mean mass of nectar sugar per flower (Nectar_mass_mean), the mean sugar concentration of nectar per flower (Nectar_conc_mean), the mass of nectar sugar per floral unit (Sugar_per_FU_ug), and the standard deviation of the mean mass (Nectar_mass_SD). Nectar_mass_SD captures the intraspecific variations in mass of nectar sugar. However, sugar concentrations were measured only in the scenario of direct measurement because of the dilution involved in the scenario of indirect measurements. All the measurements were conducted, on an average, on 18 flowers per taxon (range: 10–52). For the purpose of representativeness, flowers of different ages and sexes and across different positions on the plants and in different positions in the inflorescence were selected [25]. Collected data are presented in Table S3.

Overall, the data analyzed include 225 plant taxa in 158 genera and 55 families. Within this dataset, 157 plants are herbaceous, 63 are shrubs, and 5 are woody climbers, whereas 14 are native to UK and 211 are non-natives (see further details in [28]), showing the higher preference of humans towards non-natives than natives in their urban greenspaces.

### 4.3. Phylogenetic Tree of All the Species

I reconstructed the phylogenetic tree of all the 225 species (File S1) as implemented in the R package ‘V.PhyloMaker’ [66]. First, a species list was constructed arranged by species, genus, and family names as recommended in [66]. Then, the phylogenetic tree was reconstructed using the R function ‘phylo.maker’ using the mega-tree ‘GBOTB.extended.tre’, which is the combination of updated phylogenetic trees of previous studies [67,68].

### 4.4. Data Analysis

All analyses were run in R version 4.3.1 [69] (see File S2). First, I tested if native plants were functionally (life-forms, floral traits) different from non-natives introduced into the urban greenspaces. This test was conducted using chi-square test. Second, I investigated whether plant functional traits (life-forms, origins, and floral traits) predict nectar production. This was performed by fitting a phylogenetic ANOVA (R function phylANOVA) in library Phytools [70] to correct for the non-independence of trait values due to species shared ancestry. This approach used a combination of phylogenetic independent contrasts (PICs; [71]), and a simulation-based phylogenetic ANOVA [72] leading to Holm–Bonferroni corrected *p* values. Finally, to test if phylogeny predicts nectar production, we used the Blomberg K test [73] implemented in the R package Picante 1.2 [74]. Data analyzed for phylogenetic signal test are presented in Table S4.

## Figures and Tables

**Table 1:** Coefficients of the Blomberg K test of phylogenetic signal in nectar production based on all species (natives + non-natives). * = marginal significance, ** significance.

Nectar Production	K	PIC.var.obs.	PIC var.rnd.mean	*p*-Value	PIC.var.Z
Nectar_mass_mean	0.178063154	143,425.8953	443,998.8927	0.062	−0.384427948
Nectar_mass_SD	0.223061818	41,689.07254	193,459.6056	0.003 **	−0.55471627
Nectar_conc_mean	0.179022884	9.094378937	14.9308214	0.039 *	−1.421055868
Nectar_conc_SD	0.130033662	1.489374074	1.749948461	0.417	−0.352449477
Sugar_per_FU_ug	0.110024302	640,764.1636	1,389,605.143	0.276	−0.39843441

## Data Availability

Data analyzed are available as Supplementary Materials in this manuscript and can also be found in Ref. [22].

## Supplementary Materials

The following supporting information can be downloaded at: https://www.mdpi.com/article/10.3390/plants12183270/s1, Table S1. Coefficients of the Blomberg K test of phylogenetic signal in nectar production based on only of the subset of non-native plants; Table S2. Coefficients of the Blomberg K test of phylogenetic signal in nectar production based on only of the subset of native species; Table S3. All data collected and analyzed in the present study; Table S4. Data analyzed for phylogenetic signal test; File S1. Phylogenetic tree generated for all plants included in the present study; File S2. R script used for all conducted analyses.

## References

[1] Hulme P.E. (2009). Trade, transport and trouble: Managing invasive species pathways in an era of globalization. J. Appl. Ecol..

[2] Yessoufou K., Ambani A.E., Elansary H.O., Gaoue O.G. (2021). Alien woody plants are more versatile than native, but both share similar therapeutic redundancy in South Africa. PLoS ONE.

[3] van Kleunen M., Xu X., Yang Q., Maurel N., Zhang Z., Dawson W., Essl F., Kreft H., Pergl J., Pyšek P. (2020). Economic use of plants is key to their naturalization success. Nat. Commun..

[4] Richardson D.M., Pysek P., Rejmanek M., Barbour M.G., Panetta F.D., West C.J. (2000). Naturalization and invasion of alien plants: Concepts and definitions. Divers. Distrib..

[5] Bezeng S.B., Davies J.T., Yessoufou K., Maurin O., Van der Bank M. (2015). Revisiting Darwin’s naturalization conundrum: Ex-plaining invasion success of non-native trees and shrubs in southern. J. Ecol..

[6] Baker H.G., Baker H.G., Stebbins G.L. (1965). Characteristics and modes of origin of weeds. The Genetics of Colonizing Species.

[7] Lau J.A., Funk J.L. (2023). How ecological and evolutionary theory expanded the ‘ideal weed’ concept. Oecologia.

[8] Uesugi A., Kessler A. (2013). Herbivore exclusion drives the evolution of plant competitiveness via increased allelopathy. New Phytol..

[9] Goodell K., Parker I.M. (2017). Invasion of a dominant floral resource: Effects on the floral community and pollination of native plants. Ecology.

[10] Willis C.G., Ruhfel B.R., Primack R.B., Miller-Rushing A.J., Losos J.B., Davis C.C. (2010). Favorable Climate Change Response Explains Non-Native Species’ Success in Thoreau’s Woods. PLoS ONE.

[11] Pyšek P., Jarošík V., Chytrý M., Danihelka J., Kühn I., Pergl J., Tichý L., Biesmeijer J.C., Ellis W.N., Kunin W.E. (2011). Successful invaders co-opt pollinators of native flora and accumulate insect pollinators with increasing residence time. Ecol. Monogr..

[12] Johnson C.A., Dutt P., Levine J.M. (2022). Competition for pollinators destabilizes plant coexistence. Nature.

[13] Adedoja O., Erckie L., Boatwright J.S., van Wyk E., Geerts S. (2021). An invasive alien Proteaceae lures some, but not all nectar-feeding bird pollinators away from native Proteaceae in South African fynbos. Plant Biol..

[14] Vanbergen A.J., Espíndola A., Aizen M.A. (2017). Risks to pollinators and pollination from invasive alien species. Nat. Ecol. Evol..

[15] Mack R.N., Simberloff D., Lonsdale W.M., Evans H., Clout M., Bazzaz F.A. (2000). Biotic invasions: Causes, epidemiology, global consequences, and control. Ecol. Appl..

[16] Albrecht M., Padrón B., Bartomeus I., Traveset A., San-Jose L.M., Peñalver-Alcázar M., Milá B., Gonzalez-Jimena V., Fitze P.S. (2014). Consequences of plant invasions on compartmentalization and species’ roles in plant–pollinator networks. Proc. R. Soc. B Boil. Sci..

[17] Herron-Sweet C.R., Lehnhoff E.A., Burkle L.A., Littlefield J.L., Mangold J.M. (2016). Temporal- and density-dependent impacts of an invasive plant on pollinators and pollination services to a native plant. Ecosphere.

[18] Jones E.I., Gomulkiewicz R. (2012). Biotic Interactions, Rapid Evolution, and the Establishment of Introduced Species. Am. Nat..

[19] Vandepitte K., de Meyer T., Helsen K., van Acker K., Roldán-Ruiz I., Mergeay J., Honnay O. (2014). Rapid genetic adaptation precedes the spread of an exotic plant species. Mol. Ecol..

[20] Bossdorf O., Auge H., Lafuma L., Rogers W.E., Siemann E., Prati D. (2005). Phenotypic and genetic differentiation between native and introduced plant populations. Oecologia.

[21] Powney G.D., Carvell C., Edwards M., Morris R.K.A., Roy H.E., Woodcock B.A., Isaac N.J.B. (2019). Widespread losses of pollinating insects in Britain. Nat. Commun..

[22] Sutherland G.D., Gass C.L. (1995). Learning and remembering of spatial patterns by hummingbirds. Anim. Behav..

[23] Irwin R.E., Brody A.K. (1999). Nectar-robbing bumblebees reduce the fitness of *Ipomopsis aggregata* (Polemoniaceae). Ecology.

[24] Schemske D.W., Bradshaw H.D. (1999). Pollinator preference and the evolution of floral traits in monkey flowers (Mimulus). Proc. Natl Acad. Sci. USA.

[25] Chalcoff V.R., Gleiser G., Ezcurra C., Aizen M.A. (2017). Pollinator type and secondarily climate are related to nectar sugar composition across the angiosperms. Evol. Ecol..

[26] Janeček S., Chmel K., Ewome F.L., Hrubá K., Klomberg Y., Kobe I.N., Kouede R.D., Mertens J.E.J., Njie M.M., Tropek R. (2021). Differences in Nectar Traits between Ornithophilous and Entomophilous Plants on Mount Cameroon. Plants.

[27] Tew N.E., Memmott J., Vaughan I.P., Bird S., Stone G.N., Potts S.G., Baldock K.C.R. (2021). Quantifying nectar production by flowering plants in urban and rural landscapes. J. Ecol..

[28] Tew N.E., Baldock K.C.R., Morten J.M., Bird S., Vaughan I.P., Memmott J. (2023). A dataset of nectar sugar production for flowering plants found in urban green spaces. Ecol. Solut. Evid..

[29] Baldock K.C.R., Goddard M.A., Hicks D.M., Kunin W.E., Mitschunas N., Morse H., Osgathorpe L.M., Potts S.G., Robertson K.M., Scott A.V. (2019). A systems approach reveals urban pollinator hotspots and conservation opportunities. Nat. Ecol. Evol..

[30] Baldock K.C.R., Goddard M.A., Hicks D.M., Kunin W.E., Mitschunas N., Osgathorpe L.M., Potts S.G., Robertson K.M., Scott A.V., Stone G.N. (2015). Where is the UK’s pollinator biodiversity? The importance of urban areas for flower-visiting insects. Proc. R. Soc. B Biol. Sci..

[31] Dunnett N., Swanwick C., Woolley H. (2002). Improving Urban Parks, Play Areas and Green Spaces.

[32] Haq S.M.A. (2011). Urban Green Spaces and an Integrative Approach to Sustainable Environment. J. Environ. Prot..

[33] Tan P.Y., Wang J., Sia A. (2013). Perspectives on five decades of the urban greening of Singapore. Cities.

[34] Yessoufou K., Sithole M., Elansary H.O. (2020). Effects of urban green spaces on human perceived health improvements: Provision of green spaces is not enough but how people use them matters. PLoS ONE.

[35] Phogole B., Yessoufou K. (2023). A Global Meta-Analysis of Effects of Green Infrastructure on COVID-19 Infection and Mortality Rates. medRxiv.

[36] Richardson E.A., Pearce J., Mitchell R., Kingham S. (2013). Role of physical activity in the relationship between urban green space and health. Public Health.

[37] Maas J., Verheij R.A., Groenewegen P.P., de Vries S., Spreeuwenberg P. (2006). Green space, urbanity, and health: How strong is the relation?. J. Epidemiol. Community Health.

[38] de Vries S., van Dillen S.M.E., Groenewegen P.P., Spreeuwenberg P. (2013). Streetscape greenery and health: Stress, social cohesion and physical activity as mediators. Soc. Sci. Med..

[39] White M.P., Alcock I., Wheeler B.W., Depledge M.H. (2013). Would You Be Happier Living in a Greener Urban Area? A Fixed-Effects Analysis of Panel Data. Psychol. Sci..

[40] Diener A., Mudu P. (2021). How can vegetation protect us from air pollution? A critical review on green spaces’ mitigation abilities for air-borne particles from a public health perspective—With implications for urban planning. Sci. Total. Environ..

[41] Li Q. (2010). Effect of forest bathing trips on human immune function. Environ. Health Prev. Med..

[42] Vivier E., Tomasello E., Baratin M., Walzer TUgolini S. (2008). Functions of natural killer cells. Nat. Immunol..

[43] Bringslimark T., Hartig T., Patil G.G. (2009). The psychological benefits of indoor plants: A critical review of the experimental literature. J. Environ. Psychol..

[44] Angioy A.M., Desogus A., Barbarossa I.T., Anderson P., Hansson B.S. (2003). Extreme sensitivity in an olfactory system. Chem. Senses.

[45] Gibson M.R., Richardson D.M., Pauw A. (2012). Can floral traits predict an invasive plant’s impact on native plant–pollinator communities?. J. Ecol..

[46] Waser N.M. (1978). Interspecific pollen transfer and competition between co-occurring plant species. Oecologia.

[47] Brown B.J., Mitchell R.J. (2001). Competition for pollination: Effects of pollen of an invasive plant on seed set of a native congener. Oecologia.

[48] Tscheulin T., Petanidou T., Potts S.G., Settele J. (2009). The impact of *Solanum elaeagnifolium*, an invasive plant in the Mediterranean, on the flower visitation and seed set of the native co-flowering species *Glaucium flavum*. Plant Ecol..

[49] Morales C.L., Traveset A. (2009). A meta-analysis of impacts of alien vs. native plants on pollinator visitation and reproductive success of co-flowering native plants. Ecol. Lett..

[50] Yessoufou K., Daru B.H., Muasya A.M. (2015). Phylogenetic exploration of commonly used medicinal plants in South Africa. Mol. Ecol. Resour..

[51] Canavan S., Richardson D.M., Visser V., Le Roux J.J., Vorontsova M.S., Wilson J.R.U. (2016). The global distribution of bamboos: Assessing correlates of introduction and invasion. AoB Plants.

[52] Yessoufou K., Ambani A.E. (2021). Are Introduced Alien Species More Predisposed to Invasion in Recipient Environments if They Provide a Wider Range of Services to Humans?. Diversity.

[53] Lockwood J.L., Cassey P., Blackburn T. (2005). The role of propagule pressure in explaining species invasions. Trends Ecol. Evol..

[54] Blackburn T.M., Duncan R.P. (2001). Determinants of establishment success in introduced birds. Nature.

[55] Carlton J.T. (1996). Pattern, process, and prediction in marine invasion ecology. Biol. Conserv..

[56] Cruden R.W., Hermann S.M., Peterson S., Bentley B., Elias T. (1983). Patterns of nectar production and plant animal coevolution. The Biology of Nectaries.

[57] Pleasants J.M. (1983). Nectar production in *Ipomopsis aggregata* (Polemoniaceae). Am. J. Bot..

[58] Pyke G.H., Waser N.M. (1981). The Production of Dilute Nectars by Hummingbird and Honeyeater Flowers. Biotropica.

[59] Lotz C.N., Schondube J.E. (2006). Sugar preferences in nectar and fruit-eating birds: Behavioural patterns and physiological causes. Biotropica.

[60] Ornelas J.F., Ordano M., De-Nova A.J., Quintero M.E., Garland T. (2007). Phylogenetic analysis of interspecific variation in nectar of hummingbird-visited plants. J. Evol. Biol..

[61] Mitchell R.J. (2004). Heritability of nectar traits: Why do we know so little?. Ecology.

[62] Parachnowitsch A.L., Manson J.S., Sletvold N. (2018). Evolutionary ecology of nectar. Ann. Bot..

[63] Percival M.S. (1961). Types of nectar in angiosperms. New Phytol..

[64] Tavares D.C., Freitas L., Gaglianone M.C. (2016). Nectar volume is positively correlated with flower size in hummingbird-visited flowers in the Brazilian Atlantic Forest. J. Trop. Ecol..

[65] Faegri K., van der Pijl L. (1979). The Principles of Pollination Ecology.

[66] Jin Y., Qian H.V. (2019). PhyloMaker: An R package that can generate very large phylogenies for vascular plants. Ecography.

[67] Smith S.A., Brown J.W. (2018). Constructing a broadly inclusive seed plant phylogeny. Am. J. Bot..

[68] Zanne A.E., Tank D.C., Cornwell W.K., Eastman J.M., Smith S.A., FitzJohn R.G., McGlinn D.J., O’meara B.C., Moles A.T., Reich P.B. (2014). Three keys to the radiation of angiosperms into freezing environments. Nature.

[69] R Development Core Team (2023). R: A Language and Environment for Statistical Computing. https://www.r-project.org/.

[70] Revell L.J. (2012). phytools: An R package for phylogenetic comparative biology (and other things). Methods Ecol. Evol..

[71] Felsenstein J. (1985). Phylogenies and the Comparative Method. Am. Nat..

[72] Garland T., Dickerman A.W., Janis C.M., Jones J.A. (1993). Phyloge netic analysis of covariance by computer simulation. Syst. Biol..

[73] Blomberg S.P., Garland T., Ives A.R. (2003). Testing for phylogenetic signal in comparative data: Behavioural traits are more labile. Evolution.

[74] Kemel S.W., Cowan P.D., Helmus M.R., Cornwell W.K., Morlon H., Ackerly D.D., Blomberg S.P., Webb C.O. (2010). Picante: R tools for integrating phylogenies and ecology. Bioinformatics.

