# Supplementary material for: The Patterns of Intraspecific Variations in Mass of Nectar Sugar along a Phylogeny Distinguish Native from Non-Native Plants in Urban Greenspaces in Southern England
Source: Plants (Basel). 2023 Sep 14;12(18):3270. doi: 10.3390/plants12183270 (PMC10534836; doi:10.3390/plants12183270)
Supplement: Supplementary file 1 [file plants-12-03270-s001.zip › Table S2.pdf]

Table S2. Coefficients of the Blomberg K test of phylogenetic signal in nectar production based on only of the subset of native species.

Nectar production	*K*	*PIC.var.obs.*	*PIC var.rnd.mean*	*P val.*	*PIC.var.Z*
Nectar_mass_mean	0.234750487	57417.15969	41656.03293	0.782	0.407108455
Nectar_mass_SD	0.259751648	38086.619	30265.94239	0.764	0.254968904
Nectar_conc_mean	1.063868884	0.950225798	4.873753292	0.041*	-0.839266171
Nectar_conc_SD	0.723863421	0.158175171	0.583743638	0.148	-1.056267609
Sugar_per_FU_ug	0.576686451	216831.092	395538.9119	0.632	-0.352192557
