# Supplementary material for: The Patterns of Intraspecific Variations in Mass of Nectar Sugar along a Phylogeny Distinguish Native from Non-Native Plants in Urban Greenspaces in Southern England
Source: Plants (Basel). 2023 Sep 14;12(18):3270. doi: 10.3390/plants12183270 (PMC10534836; doi:10.3390/plants12183270)
Supplement: Supplementary file 1 [file plants-12-03270-s001.zip › Table S1.pdf]

Table S1. Coefficients of the Blomberg K test of phylogenetic signal in nectar production based on only of the subset of non-native species.

Nectar production	*K*	*PIC.var.obs.*	*PIC var.rnd.mean*	*P val.*	*PIC.var.Z*
Nectar_mass_mean	0.177249809	148955.944	505005.6981	0.103	-0.315507495
Nectar_mass_SD	0.223618982	41611.57921	184520.8274	0.004*	-0.532492049
Nectar_conc_mean	0.178445245	9.592412627	16.14594925	0.034*	-1.399899211
Nectar_conc_SD	0.126060589	1.594572089	1.921862754	0.422	-0.369909783
Sugar_per_FU_ug	0.106217382	640751.1571	1318424.875	0.36	-0.32747379
